# Disturbed Red Blood Cell Structure and Function: An Exploration of the Role of Red Blood Cells in Neurodegeneration

**DOI:** 10.3389/fmed.2018.00198

**Published:** 2018-07-16

**Authors:** Giel J. C. G. M. Bosman

**Affiliations:** Department of Biochemistry, Radboud University Nijmegen Medical Centre, Nijmegen, Netherlands

**Keywords:** aging, deformability, neuroacanthocytosis, neurodegeneration, red blood cell

## Abstract

The structure of red blood cells is affected by many inborn and acquired factors, but in most cases this does not seem to affect their function or survival in physiological conditions. Often, functional deficits become apparent only when they are subjected to biochemical or mechanical stress *in vitro*, or to pathological conditions *in vivo*. Our data on the misshapen red blood cells of patients with neuroacanthocytosis illustrate this general mechanism: an abnormal morphology is associated with an increase in the susceptibility of red blood cells to osmotic and mechanical stress, and alters their rheological properties. The underlying mutations may not only affect red cell function, but also render neurons in specific brain areas more susceptible to a concomitant reduction in oxygen supply. Through this mechanism, an increased susceptibility of already compromised red blood cells to physiological stress conditions may constitute an additional risk factor in vulnerable individuals. Also, susceptibility may be induced or enhanced by systemic pathological conditions such as inflammation. An exploration of the literature suggests that disturbed red blood cell function may play a role in the pathophysiology of various neurodegenerative diseases. Therefore, interventions that reduce the susceptibility of red blood cells to physiological and pathological stress may reduce the extent or progress of neurodegeneration.

## Introduction

The statement that a healthy red blood cell is essential for organismal homeostasis may sound as a truism, but this depends on the functional definition of a healthy red blood cell. There are many genetically determined, structural abnormalities in the hemoglobin chains that, in most circumstances, do not affect red blood cell integrity and do not seem to affect transport of oxygen binding and release in lungs and tissues, respectively (1). Also, many obvious deviations of the classical discoid red blood cell shape, due to inborn errors in integral membrane proteins and cytoskeletal components, have no obvious clinical implications (2). In addition, there are hardly any data indicating that physiological aging *in vivo* or *in vitro* during storage in the blood bank has a notable effect on oxygen supply of the tissues and carbon dioxide removal (3).

The gas transport capacity of red blood cells is not only determined by the characteristics of hemoglobin, but also by the capacity to regulate intracellular pH, deformability, ATP production, redox status, resistance to osmotic and mechanical stress, and recognition and removal by the immune system. The role of most of these processes emerges mainly upon recognition of their putative involvement in pathophysiological mechanisms, and in most cases their molecular details become clear only after detailed study *in vitro*.

The absence of conspicuous clinical consequences, such as hemolysis and anemia, of many structural and functional flaws under physiological circumstances indicates that the red blood cell has considerable reserves to maintain structure and function. The limits of these reserves, in addition to the resilience provided by the erythropoietic system, may be reached when red blood cells are exposed to pathological processes, such as inflammation (4). Errors that are inborn or flaws that are acquired in the circulation in critical structural, functional, or metabolic red blood cell components are likely to increase the rate at which the weakest links in these defenses are breached. For example, a decrease in the capacity to maintain phospholipid asymmetry increases the likelihood of recognition by macrophages, that is mediated by the exposure of phosphatidylserine (PS) in the outer leaflet of the red blood cell membrane. Aging renders red blood cells more susceptible to PS exposure after osmotic stress (5, 6).

Here we explore the boundaries of these reserves, how they may be breached, and their pathological implications. The starting point of this exploration is the complex of structural and functional characteristics of the aging red blood cell, that was the foundation of our study of the misshapen red blood cells that accompany the neurological problems of patients with neuroacanthocytosis.

## Red blood cell aging

Physiological aging *in vivo*, as well as aging *in vitro* during storage in the blood bank, induces changes in the red cell membrane (7), in the activity of the main metabolic pathways (8, 9), and in hemoglobin (10). These changes not only affect function by decreasing deformability (11, 12), but also lead to the appearance of signals that trigger recognition and removal by the immune system. Especially the latter process is induced by the conditions that the cells normally encounter in their journey through the circulation, such as mechanical stress, oxidation and hyperosmotic conditions (5, 13, 14). A number of pathological conditions may trigger the same changes, as exemplified by the detrimental effects of inflammatory lipases on red blood cell structure and the association between inflammation and anemia (4, 15). Thus, the biophysical, biochemical, immunological, and functional characteristics of the healthy, aging red blood cell provide us with the tools to study the red blood cell structure-function relationship in a clinically relevant context.

## Neuroacanthocytosis

Neuroacanthocytosis (NA) is a family of rare neurodegenerative disorders, that includes chorea-acanthocytosis, McLeod syndrome, Huntington's disease-like 2, and panthothenate kinase-associated neurodegeneration. Patients with NA suffer from devastating movement disorders, caused by degeneration of spinal neurons in the basal ganglia. One hallmark of NA is the presence of acanthocytes, red blood cells with thorny protrusions, in the blood, but detailed morphological analysis shows the presence of many other misshapen red blood cells as well (16, 17). The presence of acanthocytes is mostly considered as an indication that the pathways that lead to the red blood cell abnormalities are the same as those involved in neuronal degeneration. The molecular similarities between the putative mechanisms inducing acanthocytosis in red blood cell membrane organization and in neurodegeneration in patients with NA have been discussed extensively (18, 19).

In patients with NA, the degree of acanthocytosis may vary over time. There are no clues for the identity of the processes that might cause a transition of mature discocytes to acanthocytes. A recent inventory of the available data has led us to the hypothesis that red blood cells with an acanthocyte shape may already be present in the final stages of erythropoiesis, and appear into the circulation as such (20). This is supported by the observation that an artificially induced, long-term disturbance of red blood cell membrane architecture had a lasting effect on erythropoiesis and caused the appearance of acanthocytes in the circulation (21).

Recent applications of various combinations of immunochemical, (phospho) proteomic, lipidomic and metabolomic approaches have provided indications for the mechanisms responsible for the acanthocyte shape. In acanthocytes, Lyn kinase-mediated phosphorylation and phosphatidylinositol-involving signaling pathways show altered activities. These pathways regulate the interaction between the main cytoskeletal and integral membrane proteins, and may be involved in autophagy during erythropoiesis (19, 20, 22, 23). As a band 3 plays a central role in multiprotein complex formation during erythropoiesis (24), disturbance of this process is likely to affect the stability of the binding of the cytoskeleton to the band 3-based ankyrin-complex and/or the junctional complex. A band 3-centered disturbance of this binding leads to various abnormal cell shapes, varying from spherocytosis to ovalocytosis and acanthocytosis (2, 25). Therefore, the processes that are affected in NA must have very specific, but a yet unknown characteristics in order to induce the characteristic acanthocyte shape. Band 3 does not only provide high-affinity binding sites for the actin-spectrin cytoskeleton, but also for deoxyhemoglobin and for key enzymes of the glycolytic enzyme complex. This interaction plays a regulatory role in red blood cell metabolism and function (26). Metabolomic analyses indicate that NA-associated alterations in band 3-centered protein-protein interactions may also affect the metabolism of red cells (16). The effect of the latter changes on red blood cell survival or function are presently unclear.

**Figure 1:**
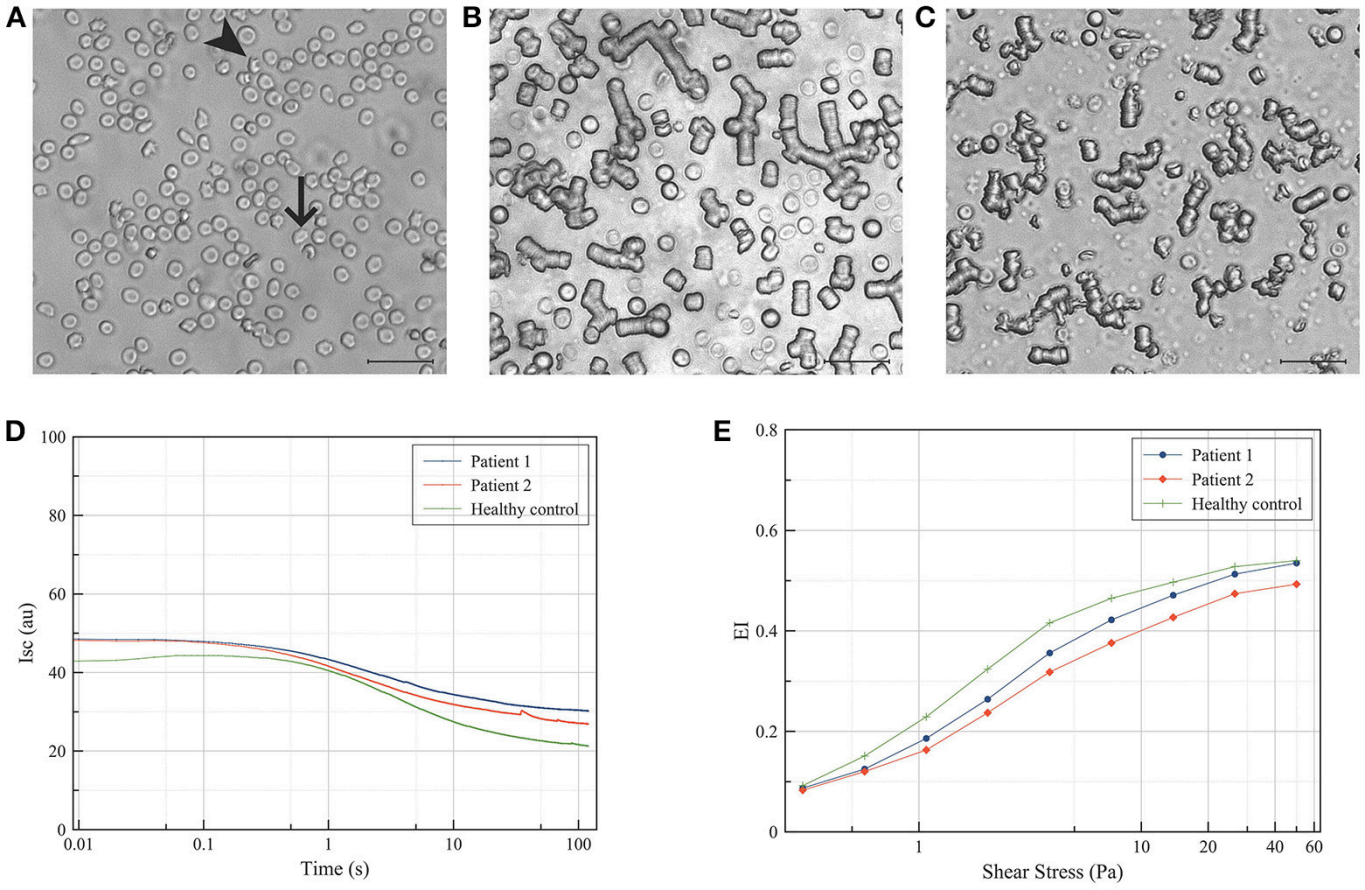
Deformability and aggregation of red blood cells from patients with neuroacanthocytosis. Red blood cells were isolated from patients with neuroacanthocytosis as described before (16), and their morphology, aggregation and deformability were compared with those of a healthy control donor. (**A)** Bright-field microscopy of the red blood cells of one patient (0.1% hematocrit in phosphate-buffered saline), showing acanthocytes (arrowhead) and otherwise misshapen red blood cells (arrow); (**B)** Bright-field microscopy of the red blood cells of a healthy control donor (1% hematocrit in plasma), showing aggregates mostly as rouleaux after 2 to 3 min of incubation at room temperature; (**C)** Bright-field microscopy of red blood cells of an acanthocytosis patient showing smaller rouleaux and much more disordered aggregates; (**D)** Syllectograms of the red blood cells of a healthy control donor and two neuroacanthocytosis patients obtained in 40% hematocrit in plasma, showing altered aggregation characteristics of the patients' red blood cells; (**E)** Deformability curves of the red blood cells of one healthy control and two neuroacanthocytosis patients, showing a lower maximum elongation index (EI) in the patients' red blood cells. Aggregation and deformability were measured using a Lorrca (RR Mechatronics, Hoorn, The Netherlands) as described before (12, 27).

Clinical descriptions of patients with NA focus on the neurological symptoms, and in general do not provide clear indications for NA-specific red blood cell dysfunction. Measurement of deformability and relaxation *in vitro* shows that acanthocytes from NA-patients assume a normal bullet-like shape when passing through a microfluidic., capillary-mimicking system, and relax toward their original shape as quickly as cells with a normal morphology. However, acanthocytes have difficulties when passing through a spleen-mimicking device *in vitro* (16). Also, the misshapen red blood cells of NA patients show a decreased deformability as well as an abnormal aggregation behavior (Figure 1). Together, these data constitute strong indications for an altered rheology and decrease in deformability, that may not only be responsible for the splenomegaly and hemolysis described in patients with McLeod disease as well as in a patient with acanthocyte-associated band 3 mutations (18, 28), but may also contribute to the neurological problems (see below).

The abnormal cytoskelon/membrane associations that underly genetically determined alterations in red blood cell morphology are, in general, associated with a decreased deformability *in vitro* (12, 29). Decreased deformability is, in most cases, associated with a decrease in hematocrit and in hemoglobin concentration *in vivo*. Even at subclinical levels, these may not only induce an increased susceptibility to red blood cell-centered pathology, as exemplified by the anemia of aging (30), but also hypoperfusion and thereby hamper oxygen delivery. In the brain, deprivation of oxygen leads to excessive glutamate release and NMDA-receptor activation-induced neuronal cell death. The latter is stimulated by Lyn-related kinases, that are also implied in acanthocyte formation during erythropoiesis and neuronal dysfunction *in vitro* (23, 31). These data, together with sporadic clinical observations, led us to the hypothesis that, in patients with neuroacanthocytosis, the compromised function of acanthocytes and otherwise misshapen red blood cells contributes to the neuronal degeneration in the striatum (20). The most likely underlying mechanism would be a decrease in red blood cell rheology, resulting in a restricted perfusion of sensitive brain areas. More subtle metabolic effects of alterations in cell morphology on oxygen binding or release by hemoglobin may play a role as well. The former mechanism may primarily be caused by defective cytoskeleton-membrane interactions, the latter by defective, membrane-centered regulation of pH, ATP production, and/or redox status.

An etiological role of acanthocytosis has been postulated in the damage to the globus pallidus and development of choreoathetosis as rare complications of cardiopulmonary bypass during open-heart surgery, especially in young children (32). In this hypothesis, the mechanical stress exerted by the extracorporeal circulation system constitutes a mechanical trigger that, in combination with hypothermia, spleen dysfunction, and/or altered pH regulation, may lead to the formation of misshapen red blood cells with a decreased deformability and to a hampered oxygen supply to the brain. A similar phenomenon may underlie the neurological problems following coronary-artery bypass surgery (33), and the higher risk of postoperative cognitive dysfunction in patients with diabetes (34). In most cases, the “postpump” chorea is transient (32). However, in NA patients a chronic acanthocytosis might lead to a chronic deficit in oxygen supply and thereby to a more severe and progressive neurodegeneration.

## Red blood cells and neurodegeneration

This hypothesis provided an additional trigger to explore the literature for indications that abnormal red blood cell function may be an etiological factor in neurodegeneration.

### Acanthocytosis

Acanthocytes are present in patients with disorders of lipid metabolism such as abetalipoproteinemia and hypolipoproteinemia. However, these patients do not have any signs of NA-like neurodegeneration, and their red blood cells have a different molecular phenotype (25, 35). Acanthocytosis has been described in patients with aceruloplasminemia, and anemia has been reported to precede neurological symptoms in almost all patiens with this defect in copper transport and iron metabolism (36, 37). These data indicate that acanthocyte generation may be due to various causes, and that the functional properties of at least some types of acanthocytic red blood cells may contribute to the development of specific neurological deficits.

### Anisocytosis

Abnormally shaped red blood cells display an increased heterogeneity in cell volume, due to impaired erythropoiesis or to excessive fragmentation or destruction. This heterogeneity, expressed as an increase in red blood cell distribution width (RDW), is associated with ischemic cerebrovascular disease (38), with increased odds of having dementia (39), with Alzheimer disease (40), and with the severity of leukoaraiosis (41). In related studies, we found indications for disturbed red blood cell aging, which is associated with changes in cell morphology, in patients with beginning demantia (42). Also, abnormal red blood cells were reported to be associated with cognitive performance in a large longitudinal aging study (43). Such associations may reflect the expression at different organs of a common pathological process. Alternatively, the abnormally shape of red blood cells in individuals with an increased RDW is likely to affect not only cellular deformability and thereby oxygen delivery (29), but may also be an indication for impaired red blood cell signaling-mediated vasodilation by NO, ATP and adenosine (44). In addition, correlations between RDW and sedentary behavior, and between RDW and muscle strength suggest that RDW may be a component of frailty in the elderly (45).

A closer look at red blood cell abnormalities in patients with various neurodegenerative diseases yields indications for abnormal cell morphology and/or red cell function in patients with Huntington's disease (46–48), Parkinson's disease (49), and Alzheimer's disease (50). These abnormalities may reflect peripheral phenomena of the major neurodegenerative mechanism, as indicated by the increased concentration of the *PARK7*-coded protein DJ-1 in red blood cells of early-stage Parkinson's disease patients (51) or by the alfa-synuclein levels in red blood cells with Parkinson's disease (52). Independent of the underlying mechanisms, the effects of these abnormalities on red blood cell function may constitute a risk factor, as has recently be argued for Alzheimer's disease (53).

### Red blood cell-centered diseases and neurological problems

Various red blood cell-centered diseases have been reported to be associated with neurological problems. In patients with sickle cell disease and thalassemia, impaired cognitive and neuropsychological functioning are likely due to inadequate oxygen supply in the frontal, parietal and temporal lobes (54–56). In these hemoglobinopathies, decreased deformability and increased aggregation are likely to be the primary causes of the neurological problems. Also, some hereditary red blood cell enzymopathies that are accompanied by hemolytic anemia are associated with neurological problems (57). The latter may be due to the expression of the same mutated genes in the brain and in hematopoietic stem cells, but also to a functional impairment of the mature red blood cells.

In addition, treatment of anemia with red blood cell concentrates, especially in transfusion-dependent patients, may pose its own problems due to its effect on perturbed iron homeostasis, also in the brain [e.g., (58)]. The molecular interplay between red blood cell homeostasis, chronic transfusion and brain pathology remains to be established.

## Conclusions

The data presented here indicate that physiological and pathological circumstances may affect red blood cell function, especially by diminishing their capacity to withstand pathophysiological stress conditions. In other words, in normal conditions, the characteristics of aging, stored, and genetically affected red blood cells may have only subclinical consequences. However, during periods of stress, for example during inflammation, already compromised cells may become less deformable, more fragile, or more prone to recognition by the immune system.

Our data on acanthocytosis illustrate that an abnormal red cell structure increases the susceptibility of the misshapen red cells to mechanical stress and alters their rheological properties. The underlying mutations may not only affect red cell shape and function, but also render neurons in vulnerable brain areas more susceptible to a concomitant reduction in oxygen supply.

Thus, interventions that reduce the susceptibility of red blood cells to pathological as well as physiological stress conditions may reduce the extent and/or progression of neurodegeneration.

## Ethics statement

The data shown here were obtained in a study that was approved by the Medical Ethical Committee of the Radboud University Medical Center and in accordance with the Declaration of Helsinki.

## Author contributions

The author confirms being the sole contributor of this work and approved it for publication.

### Conflict of interest statement

The author declares that the research was conducted in the absence of any commercial or financial relationships that could be construed as a potential conflict of interest.
